# Understanding the Relationship between Fetal Alcohol Spectrum Disorder (FASD) and Criminal Justice: A Systematic Review

**DOI:** 10.3390/healthcare10010084

**Published:** 2022-01-02

**Authors:** Francesco Sessa, Monica Salerno, Massimiliano Esposito, Nunzio Di Nunno, Giuseppe Li Rosi, Salvatore Roccuzzo, Cristoforo Pomara

**Affiliations:** 1 Department of Clinical and Experimental Medicine, University of Foggia, 71122 Foggia, Italy; francesco.sessa@unifg.it; 2 Department of Medical, Surgical and Advanced Technologies “G.F. Ingrassia”, University of Catania, 95121 Catania, Italy; monica.salerno@unict.it (M.S.); massimiliano.esposito91@gmail.com (M.E.); 3 Department of History, Society and Studies on Humanity, University of Salento, 73100 Lecce, Italy; nunzio.dinunno@unisalento.it; 4 Department of Law, Criminology, Magna Graecia University of Catanzaro, 88100 Catanzaro, Italy; lirosigiose@gmail.com; 5 Department of Biomedical and Dental Sciences and Morphofunctional Imaging, University of Messina, 98121 Messina, Italy; salvatore.roccuzzo.medicolegale@gmail.com

**Keywords:** fetal alcohol spectrum disorder (FASD), fetal alcohol syndrome (FAS), criminal justice, brain impairment

## Abstract

Prenatal alcohol exposure is considered one of the main causes of preventable birth disorders; however, it represents the main form of developmental delay in the world. Among the so-called secondary disabilities related to fetal alcohol spectrum disorder (FASD), there is a close connection with criminal behavior. This systematic review aims to provide up-to-date information about the relationship between FASD subjects and criminal justice analyzing different aspects. In light of the results of this review, a further goal is to provide several suggestions in order to reduce the public cost impact of FASD. A systematic review of the literature was conducted according to the PRISMA guidelines, producing 20 articles that met the inclusion criteria. Based on the results published in the selected studies, fetal alcohol syndrome (FAS) is a leading cause of preventable birth disorders and developmental disabilities in newborns. Moreover, these subjects seem to be more inclined to criminal acts compared to others. In conclusion, it should be pointed out that FASD entails high public health costs, both regarding the support measures provided to the affected individual and to their family, as well as the cost and social impact of any criminal offenses committed.

## 1. Introduction

Fetal alcohol spectrum disorder (FASD) is an umbrella term which includes a wide range of neurological and behavioral problems that can affect a person who was born to a woman who had abused alcohol during pregnancy [1].

The prevalence of FASD in different countries correlates with the percentage of women who drink alcohol during pregnancy. Globally, the prevalence of fetal alcohol syndrome (FAS) in the general population has been estimated at 14.6 per 10,000 [2]. Based on a recent study conducted by Popova et al. [2] the prevalence of FAS in the United States is 14.8 per 10,000, while in Canada it is 10. In Europe, the prevalence is higher in Eastern European countries that are known for their high alcohol consumption: FAS is 46.6 in Belarus, 36.5 in Russia, 34 in Ukraine, 32.7 in Bulgaria and 32.3 in Lithuania. Moreover, several European countries have a similarly alarming situation: Ireland (60.4), Denmark (45.8), United Kingdom (41.3) and Italy (33.1). Worldwide analogous data have also been registered in Australia (35.6). On the contrary, and as proof of the very close correlation between this habit and disease, FASD is a rare disease in Middle Eastern countries with prevalence rates well below 1 sick infant per 10,000 healthy ones. The countries with the lowest prevalence are Oman, the United Arab Emirates, Saudi Arabia, Qatar and Kuwait [2].

Prenatal alcohol exposure is considered one of the main causes of preventable birth defects; however, it represents the main form of developmental delay in the world [3].

An early diagnosis of FASD can improve the patient’s outcome, for example, preventing the development of secondary disabilities and helping them to lead a near-normal life, in relation to the severity of the disorder. Although it is very difficult to diagnose FASD, based on published guidelines, FAS diagnostic signs are based on different appraisements: evaluation of intrauterine or postnatal growth deficiency (height and/or weight at less than the 10th percentile); evaluation of craniofacial dysmorphology (smooth philtrum, thin vermilion and small palpebral fissures); evaluation of central nervous system damage; evaluation of prenatal alcohol exposure (confirmed, unknown, or disconfirmed) [4]. Moreover, we strongly encourage the application of a diagnostic algorithm for FASD, as described by Cook et al. [5]. Furthermore, considering that the most important causes of FASD are related to different social factors (for example low income or not well integrated subjects are more exposed to FASD risks compared to other no FASD subjects), it is important to note that further studies are needed to systematically analyze the interaction among prisons risks, FASD and different social factors, such as economic status [6].

Using coaching and support in normal daily activities can undoubtedly help subjects with FASD to find a job and live independently. Unfortunately, however, most cases of FASD remain unrecognized and, thus, diagnosis is delayed [7].

In similar cases, it is well known that late support, due to a missed or delayed diagnosis, makes the consequences of FASD irreparable. Therefore, these subjects who suffer from damage to the central nervous system caused by prenatal exposure to alcohol, will manifest a range of disabilities, from social problems to, in the most serious cases, impairment of the ability to make decisions and self-determination and to discern the social value of their behavior, associated with more or less serious antisocial and criminal behavior. These problems associated with FASD are classified as secondary disabilities, which could be improved through a range of appropriate interventions [3,8,9].

In particular, among the so-called secondary disabilities, there is a close correlation between subjects with FASD and criminal offenses [10]. Indeed, it is believed that individuals with FASD are overrepresented in correctional facilities: as reported in a study performed in the USA, 60% of adolescents and adults with FASD had been in trouble with the law, while conflicting data were obtained from a Swedish study [11]. Furthermore, when analyzing the data for adult populations, the incidence of prisoners with FASD declines dramatically [10]. It should be pointed out that FASD entails high public costs both for healthcare and the juridical system. On the one hand, there are the costs of the health measures to support the affected subjects and their relatives; on the other hand, there are the costs related both to the trial and to the social impact of the criminal act [12,13,14]. A study conducted by Canadian researchers in 2009 estimated the annual costs associated with FASD at the individual level to be CAD 21,642 [15].

This systematic review aims to provide up-to-date information about the relationship between FASD subjects and criminal justice analyzing the following aspects: FASD subjects and brain impairment; FASD subjects in indigenous groups; FASD subjects and criminal justice. In light of the results of this review, a further goal is to provide several suggestions to reduce the public cost impact of FASD, reducing the expenditure both in healthcare and in the juridical systems.

## 2. Materials and Methods

A systematic review of the literature was conducted according to the PRISMA guidelines [16].

SCOPUS, Medline (via PubMed) and Web of Science (WOS) were used as the search engine from 1 January 2001 to 1 October 2021, with the following keywords: (FASD) AND (prison); (FASD) AND (criminal); (FASD) AND (forensic); (FASD) AND (medico-legal).

### 2.1. Inclusion and Exclusion Criteria

All articles that matched the study keywords “FASD and criminal justice” were evaluated: all articles that analyzed data about subjects affected by FASD involved in criminal behavior were included. Moreover, the selection of studies was performed evaluating the characteristics of the published paper. Based on older international guidelines [4,5], during the revision process, we have included the articles that have used following acronyms: FASD; Fetal alcohol syndrome (FAS); Partial FAS (pFAS) [17]; Alcohol-Related Neurodevelopmental Disorder (ARND [18]; and Alcohol-Related Birth Defects (ARBD) [19]. Retrospective and interviewed-based studies were included. The following exclusion criteria were used: (1) review, (2) articles not in English, (3) editorial, (4) book chapter and (5) communications at conferences. The inclusion criteria were as follows: (1) original article, (2) case report, (3) articles in English, (4) in vivo studies.

### 2.2. Quality Assessment and Data Extraction

F.S initially evaluated all the articles, evaluating the title, the abstract and the whole text. M.S. then reanalyzed the articles chosen independently. In order to evaluate the degree of agreement between the studies, Kappa’s statistical test was applied [20] showing a high value κ = 0.85. Disagreements concerning eligibility were resolved by a consensus process with the supervision of C.P.

### 2.3. Risk of Bias

Different strengths are present in this systematic review, these include the amount and breadth of the studies, which span the globe, the search technique and a flowchart that describes in detail the study selection process. Moreover, the agreement between studies gave a high value for Kappa’s statistical test. The bias risks are related to the relative keywords that may have influenced the search strategy.

### 2.4. Characteristics of Eligible Studies

**Figure 1:**
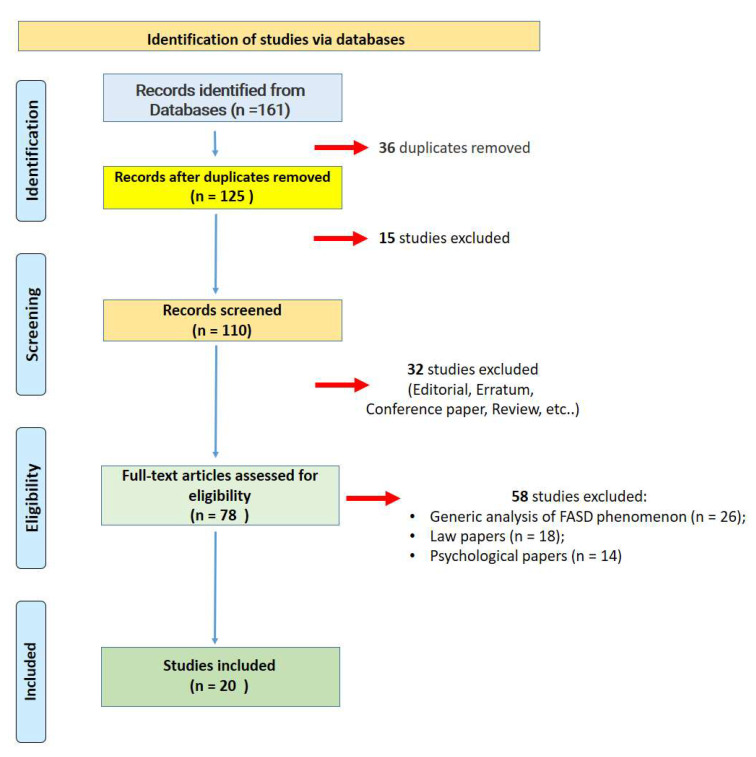
Flow diagram illustrating included and excluded studies in this systematic review.

A total of 161 articles were selected. Of these, 36 duplicates were removed and 15 studies were excluded because they did not match the study aims. Thirty-two papers were removed because they did not meet the inclusion criteria. From a total of 78 articles, 58 studies were further excluded after a careful evaluation. At the end of the review processes, 20 studies were included in the present systematic review (Figure 1).

## 3. Results

All selected articles are summarized in Table 1. Analyzing the data obtained, 12 studies were performed in Canada, 3 in Australia, 2 in the USA, and 1 study each in New Zealand, Brazil and Sweden.

### 3.1. FASD Subjects and Brain Impairment

Based on the results published in the selected studies, FAE represents a leading cause of preventable birth defects and developmental disability in newborns. As reported by Streissguth et al. [21], subjects with FAS or FAE showed arithmetic disability and specific problems with adaptive behavior. Similar results were reported by McLachlan et al. [26] who described an impairment in at least one psycholegal ability and brain impairment in FASD subjects. Banerji and Shah [27] in an FASD group reported learning disabilities and behavioral problems, developmental delay, attention deficit hyperactivity disorder (ADHD), alcohol abuse and problems with the justice system. Kambeitz et al. [35] reported that subjects with FASD were more likely to have adverse childhood events (ACEs). Increased ACEs were associated with increased rates of neurodevelopmental disorders for subjects with FASD.

Although Mela et al. [36] described the FASD group as showing clinically significant impairments in visual memory (delayed and immediate recall), with additional pronounced deficits in full-scale IQ, verbal IQ, working memory and processing speed, they reported contradictory results concerning FASD and crime involvement. It was reported that subjects in the FASD group committed fewer total crimes than those in the No-FASD group, although no statistical differences were reported. All other studies confirmed the theory that subjects exposed to alcohol in the prenatal period are more inclined to criminal activities compared to others with no FASD. Analyzing FAS subjects, Salmon and Buetow [24] reported mental health problems from alcohol and recreational drug usage: this situation is strictly related with their physical status, with a sufferance of anxiety relief, boredom, addiction and impulsivity. They described that only a few participants became involved with the justice and/or the legal system. A deficit in IQ was reported by Mullay et al., who described IQ values <70 in the FASD group [37]. These data were confirmed by Brownell et al. [34]. Finally, Tait et al. [28] reported highlighted the importance the importance of support programs to guarantee the long-term stability for individuals living with FASD.

### 3.2. FASD Subjects in Indigenous Group

Two studies reported differences in FASD exposure analyzing the data about indigenous subjects compared to non-indigenous subjects: in both studies, it emerged that indigenous subjects have a greater exposure to FAS compared to the non-indigenous group. Rojas and Gretton [23] analyzed the Canadian situation, concluding that aboriginal youth were more likely to have background histories of FASD, substance abuse, childhood victimization, academic difficulties and instability in their living environment than non-aboriginal youth. The risk of having a history of FASD in the aboriginal population is related to their social context: particularly, from a historical point of view, they have been subjected to cultural oppression, social marginalization and governmental control (living in reservations, integrating in schools and as a result of federal legislation) [40,41,42]. The importance of socio-economic factors in FASD risks was highlighted by Rangmar et al. [11] and Brownell et al. [34]: subjects in the FAS group had a lower income than those in the comparison group.

### 3.3. FASD Subjects and Justice System

A direct link between FASD subjects and problems with the justice system was described by Bower et al. [29], who confirmed a high prevalence of FASD and severe neurodevelopmental impairment in a representative sample of young subjects in detention. Similar results were obtained in two studies performed in Canada: both research groups confirmed that FASD should be considered a risk factor for problems with the justice system [31,32]. Brownell et al. [34] reported that, although FASD subjects had similar health service use to non-FASD subjects, they were more likely to be charged with a crime. Hashmi et al. [39] confirmed these data analyzing legal databases: they reported that 36% of cases dealt with offenders who had a confirmed comorbid diagnosis of FASD and the crime that was more frequently found was sexual offense. Contrariwise, Momino et al. [25] concluded that criminal behavior is determined by complex interactions between environmental and social issues, including prenatal alcohol exposure. Controversial results were obtained by Rangmar et al. [11]: this group reported a higher percentage of offenders in the FASD group; these findings were not found if the analysis was limited to subjects who had been in state care as children.

Another important aspect that may be related to FASD is imputability. In Italy, the penal code (art. 85) establishes that a person cannot be punished for a crime if when he/she committed the fact, he/she was not responsible, that is, he/she was not able to understand what he/she was doing: as in the case of FASD. Given the variations across legal jurisdictions, this issue warrants analysis at both national and international levels. Cockram [22] reported that offenders with an intellectual disability were given a custodial sentence 11.3% of the time, while those offenders without an intellectual disability received custodial sentences 8.9% of the time. Flannigan et al. [33] described young offenders with FASD displaying a profile of neurocognitive functioning more severely impaired than young offenders without FASD, analyzing numerous tasks. In this way, FASD status should be taken into consideration during trials. This important theme is remarked by Hamilton et al. 2020 [38], who underlined the importance of FASD diagnosis in subjects in prison in order to have a right process. Moreover, as reported by McLachlan et al. [30], several interventions, such as Structured Assessment of Violence Risk in Youth (SAVRY) and the Youth Level of Service/Case Management Inventory (YLS/CMI), could be useful in predicting recidivism in youths with FASD having problems with the justice system. Despite these findings, the presence of FASD is usually completely ignored by the police, as described by Salmon and Buetow [24].

## 4. Discussion

Analyzing data worldwide, as summarized in Table 1, Canada is the country that has applied great effort towards analyzing the FAS problem. Indeed, several important studies have been performed that have increased our knowledge about this important health concern. As described above, FAS is diffused in different European countries, while only Sweden has published a scientific paper on this theme. It is very strange that different countries such as Ireland, Denmark, United Kingdom and Italy have not published studies on this theme: in our opinion, in the near future, it will be important to perform new studies in order to evaluate the European situation, country by country, suggesting possible supporting actions. As recently described by Brown et al. [43], the most common consequences detected in subjects with FASD were impulse control problems, executive function deficits, learning disabilities, adaptive functioning deficits, social skill deficits and poor judgment. Based on previous studies, an early diagnosis of FASD is associated with more positive outcomes including a reduced amount of contact with the criminal justice system (CJS) [44]. Moreover, it is important to highlight that FASD may be related to cases of sudden cardiac death in infants [45].

From a forensic point of view, there are two main questions: the legal responsibility of the mother in FASD cases after alcohol consumption during pregnancy and the imputability of an FASD subject. Brown et al. [46] suggested that when rationality is impaired by FASD, acts driven by strong emotion and urges can occur, suggesting that the obvious implications regarding criminal responsibility should be reduced.

In this context, considering the responsibility of the mother, the identification of new molecular biomarkers to demonstrate prenatal exposure remains challenging for the scientific community [47,48,49,50,51]: in different countries, there is a legal responsibility for the mother if her newborn tests positive for abused substances [52]. The considerations about the mother’s responsibility are strictly related to each country. In some parts of the USA, the mother may be criminalized for drinking in pregnancy as a form of child abuse. This leads to the mother being incarcerated and the child removed from the mother’s care upon birth [53]. Contrariwise, there are also some countries (such as Canada) that do not legally determine the fetus as a human for purposes of law until birth [54]. Moreover, different initiatives should be put in place to prevent alcohol consumption during pregnancy. In Canada, Rasmussen et al. [55] described the use of the First Steps program (modeled after the Parent-Child Assistance Program) demonstrating an improvement in outcomes among women at risk for giving birth to a child with FASD. In Australia, several initiatives have been adopted, such as the presence of a court empowered to order that the mother be taken into care pending birth, or otherwise impose conditions upon conduct, with the purpose to take care of the life and health of unborn children [56]. Furthermore, as recently remarked, health institutions should organize programs to facilitate health counselling on alcohol consumption during pregnancy, particularly when providing care to women with low levels of education [57].

FASD is relevant across the legal spectrum from offensive behavior and arrest through the entire adjudication process to incarceration. For example, brain damage in FASD may be relevant, reducing the individual’s self-control and ability to recognize when his/her conduct is subjecting others to harm. The diagnosis of FASD could be very important in order to establish the sentence: after arrest, cognitive impairments may affect competency to proceed to trial by causing significant misunderstanding about the implications of waiving rights to silence and legal counsel before and during police questioning [58]. As reported by McMurtrie [59], it is important to note that subjects with FASD are treated differently by the criminal justice system in sex offense prosecutions, in relation to their role in the crime, if they are victims or perpetrators. The primary and secondary disabilities associated with FASD are usually taken into consideration when assessing the capacity of a victim to consent to sexual activity, but generally not considered in determining whether a defendant had the *mens rea* to engage in criminal sexual conduct. A comprehensive medico-legal report, prepared by professionals experienced with FASD, can help courts and attorneys to understand the complex interactions between brain injury, genetics and the environment. Corrections and probation officers should comprehend the significance of FASD and how it impacts the offender’s ability to understand and follow probation rules and orders [60].

Considering the data obtained through this literature review, the absence of a timely diagnosis of FASD may lead to serious consequences by denying these subjects the appropriate support: in this way, they cannot reduce their difficulties in carrying out daily activities; thus, there is the possibility of misbehavior. In order to reduce the adverse effects of FASD, it would be desirable to carry out timely neurodevelopmental assessments, giving appropriate support for FASD subjects. This phenomenon should be urgently analyzed jointly by national health and justice systems: undoubtedly, a timely intervention supporting FASD subjects may guarantee a lower economic burden for both the public health and juridical systems [14,61,62].

As stated above, FASD status may be considered as a risk factor for committing a criminal act, it is important to implement support measures both to prevent and to avoid recidivism by these subjects. In a recent paper, it was reported that support of health professionals is very important in order to reduce alcohol consumption during pregnancy: informing pregnant women about the harmful effects of alcohol consumption is a very important countermeasure to prevent FASD [63]. Finally, it is important to guarantee juridical support for all subjects with FASD involved in a crime. It cannot be ignored that these subjects may experience inequalities at all stages of the justice system, for example, the inability to fully understand the situation at hand.

In light of these considerations, the following activities should be adopted by each country, particularly in the case of the presence of a high rate of FASD subjects.

In our opinion, it is necessary:

- to adopt specific training for police, courts staff, lawyers and other stakeholders in order to illustrate the complex FASD phenomenon;
- to constitute a multidisciplinary group to promptly identify subjects with FASD to conduct a fair trial;
- to carry out research studies both to better define the possible incidence of FASD in the prison population and to establish targeted support interventions in prison to prevent recidivism;
- to activate a follow-up for all prisoners diagnosed with FASD.

## 5. Conclusions

As previously discussed, young subjects with FASD are at risk of poor health, education and social outcomes, having greater than normal problems with the justice system. This review stresses the usefulness of appropriate interventions to support children with FASD. Another important aspect related to FASD is the economic burden: although the data are scarce and the existing estimates likely underestimate the full economic impact of this disorder on the affected subjects, their caregivers and society, it seems to be demonstrated that FASD is a serious public health problem associated with tremendous economic burden. A reduction in the societal costs of FAS, both preventive and targeted interventions for children with FAS, should be prioritized. For example, different countermeasures should be applied by governments, providing services to educate and train subjects with FASD, their family members, teachers and social service workers to prevent victimization and report victimization when it occurs. Moreover, in the same manner, the justice system and law enforcement should develop systems to protect the rights of subjects with FASD who are victimized, especially when they appear as witnesses.

Part of changing the landscape of FASD is to modify the way the community perceives and reacts to FASD. A multidisciplinary approach to addressing this problem is increasingly necessary. Rather than a purely psychological or medical approach, the integration of FASD subjects is the most important measure that can be taken to prevent the risk of criminal behavior. This becomes a way to change the conversation around FASD from the current sense of certain failure to one of possibility of success.

Finally, well-designed studies should be performed, country by country, to provide an up-to-date status, allowing each government to adopt new and useful interventions.

## Figures and Tables

**Table 1:** Summary of the details of the systematic review.

Reference	Country	Study	Main Findings
Streissguth et al., 2004 [21]	USA	Sample: 415 subjects including 155 with FAS and 260 with fetal alcohol exposure (FAE) were enrolled: 60% white, 25% Indigenous American, 7% Black, 6% Hispanic. Diagnosis: Diagnoses were by dysmorphologists. Methodology of the study: Life History Interview administered by telephone. Economic status: No data were reported.	Psychological tests in FAS and FAE subjects revealed two main deficits: specific arithmetic disability and specific adaptive behavior problems, evaluating criminal behavior.
Cockram, 2005 [22]	Australia	Sample: group of offenders with intellectual disability (843). Aboriginal 8.3%. Diagnosis: not reported. Methodology of the study: Analysis of Disability Services Commission (DSC) database. Economic status: No data were reported.	This study shows that subjects with an intellectual disability received a prison sentence in 11.3% of cases, while offenders without an intellectual disability received a prison sentence in 8.9%.
Rojas and Gretton, 2007 [23]	Canada	Sample: Aboriginal (n = 102) and non-Aboriginal (n = 257) youth who engaged in sexual offending behavior and were ordered to attend a sexual offender treatment program were enrolled. FASD subjects (n = 63) Diagnosis: Diagnoses based on database data, analyzing participants’ discharge from a Youth Sexual Offence Treatment Program (YSOTP). Methodology of the study: Analysis of database. Economic status: No data were reported	This study shows that Aboriginal youth were more likely to be affected by FASD, substance abuse, child victimization, academic difficulties and instability in the living environment. Aboriginal youth were significantly more likely than non-Aboriginal youth to have a living situation rated as unstable.
Salmon and Buetow, 2012 [24]	New Zealand	Sample: 14 subjects (6 Maori, 6 New Zealanders European, 1 America, 1 Cook Island) with FAS were enrolled. Diagnosis: professionally diagnosed Methodology of the study: interview (audio-recorded, face-to-face in-depth, unstructured questioning was used). Economic status: Concerning their economic status, 6 were not employed, 2 employed, 6 no answer.	All participants reported mental disorders due to the use of alcohol and recreational drugs. Some participants were involved with the justice and/or the legal system. FASD was considered during the judicial phases.
Momino et al., 2012 [25]	Brazil	Sample: 262 male adolescents institutionalized because of criminal behavior (alcohol use admitted by 48.8% of the mothers) and 154 male students (alcohol use admitted by 39.9% of the mothers) were enrolled. Diagnosis: FAS diagnostic signs were defined according the guidelines of the Institute of Medicine. Methodology of the study: A questionnaire was completed by the mother or by the legal guardian. Economic status: No data were reported.	The results of this study showed that criminal behavior was more pronounced in the FAS group, although this was influenced by complex environmental and social interactions, including prenatal exposure to alcohol.
McLachlan et al., 2014 [26]	Canada	Sample: Two groups of young offenders (50 with FASD and 50 without prenatal alcohol exposure) Diagnosis: professionally diagnosed Methodology of the study: Questionnaire. Economic status: No data were reported.	The findings showed that a large number of young offenders with FASD (90%) demonstrated impairment in at least one psychological skill and the rates of impairment were significantly higher than in the comparison group.
Rangmar et al., 2015 [11]	Sweden	Sample: Data of 79 subjects with FAS were compared with Control Group (n = 3160). Diagnosis: Diagnoses were performed based on literature indication. Methodology of the study: Database (national register–based study) analysis. Economic status: About 82% of subjects with FAS had a disposable income in the 3 lowest quintiles.	Analyzing the criminal acts committed in the two groups, 27.8% of the subjects in the FASD group had at least one record of a judicial conviction (20.3% in the control group) and 6.3% had been convicted of a serious crime (4.0% in the control group).
Banerji and Shah, 2017 [27]	Canada	Sample: 49 children with FASD were enrolled in this study Diagnosis: The diagnoses of FASD were based on the 2005 Canadian guidelines. Methodology of the study: Interview and database analysis (interviews with the biological mother, review of medical or social service records). Economic status: No data were reported.	Subjects with FASD demonstrated various problems such as learning difficulties and behavioral problems, developmental delay, attention deficit hyperactivity disorder (ADHD), alcohol abuse. Moreover, 6/49 were involved with the criminal justice system.
Tait et al., 2017 [28]	Canada	Sample: Two male psychiatric patients with FASD with criminal issues Diagnosis: The diagnoses of FASD were based on the 2005 Canadian guidelines. Methodology of the study: Follow-up during life. Economic status: No data were reported.	The authors underlined the importance of support programs in order to guarantee the long-term stability for individuals living with FASD.
Bower et al., 2018 [29]	Australia	Sample: 99 young subjects were included in this study: 88 subjects had at least one domain of severe neurodevelopmental impairment and 36 were diagnosed with FASD; 73 are Aboriginal. Diagnosis: Australian diagnostic criteria were applied. Methodology of the study: Face-to-face approach. Economic status: No data were reported.	This study confirmed that subjects with FASD show severe neurodevelopmental disorders.
McLachlan et al., 2018 [30]	Canada	Sample: 100 justice-involved youth were enrolled, including 50 diagnosed with FASD and 50 without FASD or prenatal alcohol exposure. Diagnosis: Not reported. Methodology of the study: Interview and database analysis. Economic status: No data were reported.	The results of this study support the validity of violence risk assessment tools (such as the Structured Assessment of Violence Risk in Youth and the Youth Level of Service/Case Management Inventory) in predicting recidivism in justice-involved young subjects with FASD.
McLachlan et al., 2019 [31]	Canada	Sample: 80 justice-involved adults were enrolled. Diagnosis: the 2005 Canadian Diagnostic Guidelines for FASD with the support of FAS facial photographic analysis software. Methodology of the study: Interview, using semi-structured medical and social history interview. Economic status: No data were reported.	In the sample analyzed, the authors identified about 17% of the subjects with FASD; moreover, about 31% of subjects had been exposed to alcohol in the prenatal period.
Brintnell et al., 2019 [32]	Canada	Sample: 49 subjects (Caucasian (37%) and Indigenous (57%), with 12% of Indigenous participants identified with specific First, Nation groups and 6% whose ethnicity was unknown) with FADS were recruited. Diagnosis: neuropsychological testing and a psychiatric interview Methodology of the study: Test and Interview. Economic status: No data were reported.	The sample analyzed confirms the finding that FASD is a risk factor for criminal behavior.
Flannigan et al., 2019 [33]	Canada	Sample: The authors compared two groups: FASD (38) vs non-FASD (43). Diagnosis: not reported. Methodology of the study: Database revision. Economic status: No data were reported.	Based on numerous parameters analyzed, subjects with FASD displayed a severely impaired neurocognitive functioning profile compared to the control group.
Brownell et al., 2019 [34]	Canada	Sample: 1058 subjects with FASD compared with non-FASD (2229) subjects. Diagnosis: Diagnoses based on the reported data. Methodology of the study: Database analysis. Economic status: Nearly two-thirds (63%) of the FASD group was in the lowest income quintile (Q1); about one-third (29%) in the non-FASD group.	The results show that although FASD subjects had similar involvement with health services as non-FASD subjects, they were more likely to be charged with a crime, showing greater involvement with the judiciary.
Kambeitz et al., 2019 [35]	USA	Sample: The data of 98 subjects with FASD were compared with data of 105 non-FASD (controls). Diagnosis: Based on different published criteria. Methodology of the study: Database analysis. Economic status: No data were reported.	Data from the present study confirm that subjects with FASD are more likely to have adverse childhood events (ACEs) than the control group. Increased ACEs were associated with higher rates of neurodevelopmental disorders for subjects with FASD.
Mela et al., 2020 [36]	Canada	Sample: 45 subjects were included in the study from an outpatient forensic psychiatric clinic. Diagnosis: Canadian guidelines for diagnosing FASD. Methodology of the study: self-report questionnaires. Economic status: The authors reported that: 14% was employed full-time, 11.6% part-time, 7% full-time student, 23.3% sick/disability leave, 41.9% was unemployed, 2.3% retired.	The results of this study showed that subjects in the FASD group committed fewer total offenses than those in the No-FASD group, although no statistically significant differences were reported. However, significant cognitive impairment was found in the FASD group compared to the control group.
Mullaly et al., 2020 [37]	Canada	Sample: Data of FASD group (25 subjects with a confirmed or possible FASD diagnosis) were compared with data of the criminal justice (CJ) group (55 subjects without FASD). Diagnosis: 2005 Canadian Diagnostic Guidelines for FASD. Methodology of the study: Tests and data analysis. Economic status: No data were reported.	The main findings of this study showed that participants with diagnosed/possible FASD were more likely to fail a single performance validity test (PVT) and failed more PVTs overall than those without FASD. Participants in the FASD group had an IQ <70 on a standard measure of intellectual functioning compared to the control group.
Hamilton et al., 2020 [38]	Australia	Sample: 38 participants were enrolled (27 were aboriginal youths; 9 subjects with confirmed FASD diagnosis). Diagnosis: Not reported. Methodology of the study: Interview (structured, semi-structured and unstructured interviewing). Economic status: No data were reported.	The authors underlined the importance of FASD diagnosis in subjects in prison in order to have a correct trial.
Hashmi et al., 2021 [39]	Canada	Sample: 61 cases were included (44% indigenous people), 36% with FASD. Diagnosis: multidisciplinary team. Methodology of the study: Legal database analysis. Economic status: Lower socioeconomic status.	Cognitive impairment may be considered an important risk factor in order to commit a crime, particularly in the cases of sexual violence.

## Data Availability

Data sharing not applicable, no new data were created or analyzed in this study.
